# Paraoxonase 1 gene (*PON1*) variants concerning hepatitis C virus (HCV) spontaneous clearance in hemodialysis individuals: a case–control study

**DOI:** 10.1186/s12879-021-06597-4

**Published:** 2021-08-26

**Authors:** Alicja E. Grzegorzewska, Adrianna Mostowska, Wojciech Warchoł, Paweł P. Jagodziński

**Affiliations:** 1 Department of Biochemistry and Molecular Biology, Poznan University of Medical Sciences, 60-781 Poznań, Wielkopolska Poland; 2 Department of Ophthalmology and Optometry, Poznan University of Medical Sciences, 60-806 Poznań, Wielkopolska Poland

**Keywords:** HCV RNA, Hemodialysis, *IFNL4* rs368234815, *PON1* variants, Spontaneous HCV clearance

## Abstract

**Background:**

To explore associations between *PON1* rs854560, rs662, 705,379, HCV clearance, and interactions between tested *PON1* single nucleotide variants (SNVs) and interferon-λ4 gene (*IFNL4*) rs368234815 variant in hemodialyzed individuals.

**Methods:**

The study included 83 HD individuals who spontaneously resolved HCV infection (all had known *IFNL4* rs368234815 variant) and 104 individuals with persistently positive blood tests for HCV RNA (102 were *IFNL4* rs368234815 variant successfully genotyped). We genotyped *PON1* by high-resolution melt analysis (rs662) or predesigned TaqMan SNV Genotyping Assay (rs854560, rs705379). We used a logistic regression model to assess the association between genetic data and HCV outcome while adjusting for clinical confounding variables. Epistatic interactions between tested *PON1* SNVs and *IFNL4* rs368234815 were analyzed by the multifactor dimensionality reduction method.

**Results:**

In the recessive inheritance model, *PON1* rs662 GG (OR 9.94, 95% CI 1.20–82.7, *P* = 0.022) and rs854560 TT (OR 4.31, 95% CI 1.62–11.5, *P* = 0.003) genotypes were associated with a higher probability for HCV clearance. The haplotype composed of rs662A_rs854560A_rs705379 was not associated with spontaneous HCV clearance. The *IFNL4* rs368234815 TT/TT variant was equally distributed among individuals bearing different *PON1* SNVs. The epistatic gene–gene analysis did not reveal the interaction between tested *PON1* SNVs and *IFNL4* rs368234815 (*P* = 0.094). Regression model, including the *PON1* rs662 GG genotype, the *PON1* rs854560 genotype, the *IFNL4* rs368234815 TT/TT genotype, age at RRT onset, RRT duration, and chronic glomerulonephritis as possible explanatory variables for spontaneous HCV clearance, showed that significant predictors of spontaneous HCV clearance were the *IFNL4* rs368234815 TT/TT genotype (OR 2.607, 95% CI 1.298—5.235, *P* = 0.007), *PON1* rs854560 TT (OR 6.208, 1.962–19.644, *P* = 0.002), *PON1* rs662 GG (OR 10.762, 1.222–94.796, *P* = 0.032), and RRT duration (OR 0.930, 95% CI 0.879–0.984, *P* = 0.011).

**Conclusion:**

In HD individuals, *PON1* rs662 GG and rs854560 TT are associated with a higher frequency of spontaneous HCV clearance.

**Supplementary Information:**

The online version contains supplementary material available at 10.1186/s12879-021-06597-4.

## Background

Hepatitis C virus (HCV) infection is one of the most prevalent infections worldwide. An estimated 160–170 million individuals have HCV infection, 71 million of them have HCV-associated chronic liver disease [1]. The global prevalence of people older than 15 years showing antibodies against HCV (anti-HCV) is 2.0% [2].

Outcomes of HCV infection were associated with single nucleotide variants (SNVs) of interferon-λ genes (*IFNLs*), located on chromosome 19q13 (persistent HCV infection—the TT genotype of *IFNL4* rs12979860, the ∆G/∆G genotype of *IFNL4* rs368234815 [3], pegylated interferon plus ribavirin treatment-induced clearance—the TT/TT genotype of *IFNL4* rs368234815 [4], spontaneous HCV clearance—the CC genotype of *IFNL4* rs12979860, the TT/TT genotype of *IFNL4* rs368234815 [3, 5, 6], direct-acting antiviral medication-induced HCV clearance—the TT/TT genotype of *IFNL4* rs368234815, the TT genotype of *IFNL4* rs12979860 [7, 8]). The *IFNL4* rs368234815 is related to interferon-λ4 (IFN-λ4) production associated with HCV outcome [3]. Many other genetic influences were also identified or suggested, including *PON1* SNVs placed on chromosome 7q21.3 [9–11].

The *PON1* rs662 G allele (also known as R allele) codes the paraoxonase 1 (PON-1) isoform containing arginine at position 192 (B alloenzyme), which is associated with the less active antioxidant properties than that of PON-1 isoform having glutamine at this site (A alloenzyme) and related to the rs662 A allele (also known as Q allele) [12, 13]. However, opposite results were also demonstrated [14–16]. *PON1* rs662 had little impact on paraoxonase concentration [16]. The *PON1* rs854560 SNV (L55M, 163A > T) affects position 55 and involves a methionine (T allele, also known as M allele) to leucine (A allele, also known as L allele) conservative interchange. *PON1* rs854560 influences mRNA levels. The rs854560 T allele transcript related to methionine-containing PON-1 isoform is less stable than the A allele transcript coding leucine-containing PON-1 isoform. Therefore, the *PON1* rs854560 T allele is associated with a lower circulating PON-1 concentration. Circulating PON-1 activity was also shown to be decreased in the rs854560 T allele bearers, but PON-1 activity, corrected for protein, does not differ significantly between *PON1* rs854560 genotypes [16]. *PON1* rs705379 is located in the gene non-coding region within the sequence GGCGGG. This sequence is the binding site of the Sp1 transcription factor. Changes in this sequence affect the *PON1* expression. *PON1* rs705379 is involved in forming complexes between the Sp1 and the *PON1* promoter [17]. Sp1 acts as a positive regulator of PON1 transcription [18]. The binding of Sp1 to the -108 site is weaker in the presence of allele T than allele C [19, 20]. The *PON1* rs705379 CT and TT genotypes correspond with the lower PON-1 activity [21]. The *PON1* rs705379 variant is responsible for approximately 12% of the changes in PON-1 activity [22]. Therefore, the *PON1* rs705379 variant, although non-coding, influences other *PON1* SNVs and contributes to their function [20, 21].

*PON1* SNVs, engaged in the PON-1 coding, are related to antioxidant, anti-inflammatory, anti-apoptosis, anti-thrombosis, anti-adhesion, and lipid-modifying processes [23–25]. Persistent HCV infection is accompanied by increased oxidative stress [26], hepatic inflammation [27], thrombosis [28], platelet adhesion [29], and lipid abnormalities [30]. Additionally, compared to healthy controls, DNA oxidative damage in circulating leukocytes, identifiable by the 8-hydroxydeoxyguanosine formation, was higher in individuals with HCV persistent infection, also in those showing normal aminotransferase levels [31].

Individuals with chronic hepatitis C compared with healthy individuals showed a higher frequency of the arginine-containing PON-1 (RR192 isoform) associated with the *PON1* rs662 GG genotype [9]. In this study, the plasma concentration of total peroxides was higher in individuals carrying the RR isoform, but the RR homozygote individuals were most able to hydrolyze paraoxon. On the other hand, downregulation of PON-1 was suggested as an indicator for predicting the poor survival of individuals with hepatocellular carcinoma [32]. Pegylated interferon and ribavirin treatment efficacy were not demonstrated as dependent on *PON1* SNVs [10]. Taking into account *PON1* SNVs functionality, they could be relevant as candidates for investigation of HCV infection outcome.

Uremic individuals treated with hemodialysis (HD) are especially prone to HCV infection due to insufficient antiviral prevention during the repeated use of extracorporeal blood circuits for HD sessions, altered immunocompetence related to the uremic status, and a lack of prophylactic HCV vaccination. The prevalence of HCV infection in HD individuals varies worldwide from 1% to over 70%. In the United States, it is tenfold higher than in the general population [33].

The *IFNL4* variant rs368234815 was the strongest predictor of spontaneous HCV clearance among other *IFNL3/IFNL4* variants [34]. *PON1* SNVs and PON-1 activity distribution did not differ concerning HCV infection in a small group of HD individuals (20 HCV infected, 26 HCV not infected) [11].

We aim to explore associations between three *PON1* SNVs (rs705379, rs854560, and rs662) and spontaneous clearance of HCV infection in uremic individuals treated with maintenance HD. These *PON1* SNVs were the most frequently analyzed in the general population, and also in HD individuals, concerning mainly atherosclerosis-related complications [23], antioxidant activity [24], diabetes mellitus [35], and dyslipidemia [36]. Data related to HCV infection concerning *PON1* SNVs are very rare in non-uremic individuals [9, 10]. The uremic milieu of dialyzed individuals might influence the transcription and translation of *PON1* SNVs, probably downregulate, as serum PON-1 activity and concentration are usually decreased in uremia. The uremic rat study showed that among 10,153 genes with an expression level of > 1 reads/kilobase transcript/million mapped reads, 2,663 genes were differentially expressed (47% upregulated and 53% downregulated) [37]. Therefore, *PON1* SNV associations with HCV clearance could be negatively influenced by uremia. To our best knowledge, the influence of *PON1* SNVs on HCV clearance was not investigated in HD individuals. Haplotypes of *PON1* SNVs and epistatic interactions between tested *PON1* SNVs and the *IFNL4* variant rs368234815 were also investigated.

## Methods

### Design, setting of the study, and the characteristics of participants

DNA probes for *PON1* SNV genotyping (n = 1408) were received from individuals who started renal replacement therapy (RRT) between June 15, 1983, and April 29, 2019. In our genomic collection, we had 1332 individuals who had successfully genotyped *PON1* rs662, 1362 individuals with genotyped *PON1* rs854560 SNV, 1329 individuals with genotyped *PON1* rs705379, and 440 individuals with genotyped *IFNL4* rs368234815. All *PON1* and *IFNL4* genotyped individuals were not related, treated with HD, and had established HCV status.

The research was planned as a case–control study. From the entire group of 1408 individuals, we have chosen the study group composed of individuals who spontaneously resolved HCV infection (the case group) or remained persistently HCV infected (the control group).

At enrolment, the inclusion criteria for the whole study group were the treatment with maintenance HD (for comparison of data obtained with the same RRT method), no genetic relationship with other tested individuals, and stable clinical status at data collection. For the case group, the inclusion criteria additionally included stable anti-HCV (positive) and HCV RNA (negative) testing results. The control individuals had to show stable anti-HCV and HCV RNA positivity. Stable clinical status concerning viral results was recognized if viral data were unchangeable during a period from the first determination of specific phenotype to the time of the study, but not shorter than one year.

Exclusion criteria for all study groups included treatment with pegylated interferon plus ribavirin or direct-acting antiviral medication.

Demographic, clinical, and laboratory data of enrolled individuals were derived from patient files. As we update individuals` data every year, we used the latest stable results of each individual for comparisons between groups. Stable clinical and laboratory data were recognized if HD individuals were at least six weeks free from blood or plasma transfusions, as well as free from more considerable surgery and acute diseases during three months preceding enrolment.

Individuals were enrolled from dialysis centers located in the Greater Poland voivodship (Poland). The study included 83 HD individuals who spontaneously resolved HCV infection (all had known *IFNL4* rs368234815 variants) and 104 individuals with persistently positive blood tests for HCV RNA (102 were *IFNL4* rs368234815 variants successfully genotyped). The number of controls per case was 1.25.

### PON1 genotyping

*PON1* SNVs (rs705379, rs854560, and rs662) were genotyped using previously described methods [35, 36]. Genomic DNA was obtained from peripheral blood mononuclear cells. The extracted DNA was stored in -20 °C and genotyped in the Department of Biochemistry and Molecular Biology, Poznan University of Medical Sciences, the Greater Poland voivodship, Poland. Genotypes were called separately for each genotyping 96-well plate. For all SNPs, quality control was ensured by including 10% of the samples as duplicates. Samples that failed the genotyping assay were excluded from statistical analyses.

### Laboratory testing

During the DNA collection period, anti-HCV and HCV RNA testing were changing. Still, laboratory methods were always recommended by country and world epidemiological authorities as sufficient for clinical determinations (reagents had CE marketing certificates for in vitro diagnostics). In both study groups, anti-HCV was qualified by the microparticle enzyme immunoassay (Abbott Laboratories, Wiesbaden, Germany) or immunochemiluminescence (Abbott Laboratories, Wiesbaden, Germany).

The assay for HCV RNA exploited the TaqMan principle (Roboscreen, Leipzig, Germany—sensitivity for HCV RNA 173 IU/mL) or RealTime Reverse Transcription–Polymerase Chain Reaction method (Abbott Laboratories, Wiesbaden, Germany–analytical sensitivity for HCV RNA 12 IU/mL, Roche Molecular Systems, Inc., Branchburg, NJ, USA–sensitivity for HCV RNA 18.354 IU/mL or GeneProof, Brno, Czech Republic—sensitivity for HCV RNA 18.354 IU/mL). It is practically impossible that different methods of anti-HCV and HCV RNA measurements, applied in the study individuals, could influence their allocation to case and control groups, especially that the assessments were regularly repeated (every 6–12 months) in dialysis centers. HD individuals diagnosed with different methods would have received HCV RNA assessment with more sensitive assays over RRT duration.

Other laboratory parameters were determined using routine methods.

### Statistical analysis

We applied the Genetic Association Study Power Calculator (http://csg.sph.umich.edu/abecasis/gas_power_calculator/index.html) to find the expected power at genotype odds ratio of 1.00–2.50. Spontaneous HCV clearance was assumed to occur in 14%–46% of individuals [38]. In HD individuals of the Great Poland region, HCV clearance was shown in 42.2% of cases [34]. For 100 individuals who spontaneously resolved HCV infection (the case group) and 100 who remained persistently HCV infected (controls), a power of about 80% could be shown at an odds ratio of at least 1.5 (Additional file 1: Table S1).

Basic data of controls and individuals were compared using the Mann–Whitney test for quantitative variables and Fisher’s exact test for dichotomous variables. Differences between tested individuals` phenotypes in *PON1* genotype groups were analyzed using all available *PON1* SNVs. Quantitative variables were tested using the Kruskal–Wallis test with multiple mean rank comparisons as a post hoc. Dichotomous variables were tested using Fisher's exact test for 3 × 2 contingency tables. The post hoc analyses were made using Fisher's exact test for 2 × 2 contingency tables (repeated for each pair of genotypes). Phenotypes yielding significance in these analyses were subject to adjustment of genetic associations between *PON1* SNPs and spontaneous HCV clearance. An adjustment was computed by the regression analysis evaluated using Wald test with post hoc tests, if appropriate.

The Hardy–Weinberg equilibrium (HWE) was computed by comparing the observed genotype frequencies to the expected ones by the Chi-square test (P > 0.05 with df = 1 for balance). Differences between genotype and allele frequencies in tested groups were computed using Fisher's exact test. Cochran-Armitage and Fisher's exact tests were used to evaluate *PON1* SNV associations in models of inheritance.

We used a logistic multivariate regression model including selected genetic and clinical data, which significantly differed between individuals with spontaneous HCV clearance and individuals with persistent HCV infection (age at the RRT onset, RRT duration, chronic glomerulonephritis as a cause of end-stage renal disease, *IFNL4* rs368234815 TT/TT vs. ∆G/TT + ∆G/∆G, *PON1* rs854560 TT vs. AA + AT, *PON1* rs662 GG vs. AA + AG). These parameters were used as possible explanatory variables for HCV outcome. Individuals with spontaneous HCV clearance and individuals with persistent HCV infection differed also in liver enzymes activities, but these differences resulted from persistency of HCV infection, so there were considered as response variables and were not used in the logistic multivariate regression as possible explanatory variables for HCV outcome. A receiver operating characteristic (ROC) curve was computed to create the area under the curve (AUC) to measure the regression model accuracy. Fit diagnostics for the multivariable model were performed using Hosmer Lemeshow goodness of fit quality of parameters estimation and reliability (statistical significance) of the regression coefficient for the logistic regression model.

Pair-wise linkage disequilibrium (LD) between *PON1* nucleotide variants was computed. D’ and r^2^ were obtained by the Haploview 4.2 software (http://www.broad.mit.edu/mpg/haploview/). The same software calculated haplotype frequencies. Epistatic interactions between tested *PON1* SNVs and *IFNL4* were analyzed by the multifactor dimensionality reduction (MDR) method [39]. Statistical significance in the haplotype and gene–gene interactions was assessed using the 1000-fold permutation test.

P-values below 0.05 were considered statistically significant.

## Results

Table 1 demonstrates characteristics of HD individuals showing spontaneous HCV clearance and those who remained persistently HCV RNA positive. Individuals who resolved HCV infection presented higher frequency of the *IFNL4* rs368234815 TT/TT genotype, were older at RRT onset, had shorter RRT duration, less frequently demonstrated chronic glomerulonephritis as a cause of end-stage renal disease, and showed lower activities of liver enzymes.

**Table 1:** Characteristics of HD individuals concerning outcome of HCV infection

Parameter	Spontaneous HCV clearance n = 83	Persistent HCV RNA positivity n = 104	*P* value^a^
Male gender	43 (51.8)	57 (54.8)	0.768
Age at RRT onset, years	53.3 (7.2–85.9)	47.3 (8.7 –79.5)	0.011
Diabetic nephropathy	19 (22.9)	17 (16.3)	0.270
Chronic glomerulonephritis	16 (19.3)	37 (35.6)	0.015
Hypertensive nephropathy	9 (10.8)	11 (10.6)	1.000
RRT duration, years	7.12 (0.19–34.01)	13.32 (0.65–32.81)	0.002
BMI, kg/m^2^	24.43 (16.23–37.93)	23.18 (15.22–44.8)	0.165
HBsAg positivity	7 (8.4)	5 (4.8)	0.375
ALT, IU/L	12.9 (3–63)	22 (2–195)	0.0001
AST, IU/L	15 (8–50)	22 (7–152)	0.00005
ALP, IU/L	86.63 (45–803.75)	110 (34.5–647.25)	0.024
GGT, IU/L	19.7 (7–692)	39 (4–498)	0.001
C-reactive protein, mg/L	5.4 (0.3–105.5)	5.4 (0–90.7)	0.536
Albumin, g/dL	3.9 (2–36)	3.8 (1.9–38)	0.513
Platelet count, × 10^9^/L	177 (93–397)	177 (70–405)	0.353
Total cholesterol (mg/dL)	156 (98.9–280)	158 (65–259)	0.394
HDL cholesterol (mg/dL)	38 (16.5–88)	40 (13–102)	0.318
LDL cholesterol (mg/dL)	82.5 (32–186.3)	84 (17.4–174)	0.700
TG (mg/dL)	139 (36.3–479)	139 (32–419)	0.577
Non-HDL-cholesterol (mg/dL)	120.25 (52–241)	117 (22.5–225)	0.306
LDL/HDL cholesterol ratio	0.641 (0.101–1.81)	0.632 (0.084–2.875)	0.987
HDL/TC ratio	0.236 (0.111–0.505)	0.272 (0.094–0.685)	0.054
TG/HDL cholesterol ratio	3.38 (0.65–25.55)	3.29 (0.74–27.93)	0.471
TG/HDL-cholesterol ratio ≥ 3.8	29 (41.4%)	38 (40.9%)	1.000
*IFNL4* rs368234815 TT/TT	46 (55.4%)	33 of 102 (32.4%)	0.002

Results are presented as median and range (minimum–maximum) or the number of individuals presenting the indicated parameter with the % of the total of tested individuals shown in parentheses

^a^Mann–Whitney test for quantitative variables, Fisher’s exact test for dichotomous variables

Conversion factors to SI units are as follows: for alanine aminotransferase—1 IU/L = 0.0167 µkat/L, for albumin—1 g/dL = 10 g/L, for alkaline phosphatase—1 IU/L = 0.0167 µkat/L, for aspartate aminotransferase—1 IU/L = 0.0167 µkat/L, for cholesterols—1 mg/dL = 0.0259 mmol/L, for C-reactive protein—1 mg/L = 9.524 nmol/L, for gamma-glutamyltransferase—1 IU/L = 0.0167 µkat/L, for triglycerides—1 mg/dL = 0.0113 mmol/L

*ALP* alkaline phosphatase, *ALT* alanine aminotransferase, *AST* aspartate aminotransferase, *Anti-HCV* antibodies against hepatitis C virus, *BMI* body mass index, *GGT* gamma-glutamyltransferase, *HBsAg* surface antigen of hepatitis B virus, *HCV* hepatitis C virus, *HD* hemodialysis, *RRT* renal replacement therapy

Individuals stratified by *PON1* rs662 and rs854560 genotypes did not differ significantly in frequency of the tested phenotypes (Additional file 1: Tables S2 and S3). Diabetic nephropathy was more commonly found in those with the rs705379 TT genotype, while higher serum albumin concentrations were observed in those with the rs705379 CC genotype.

The distribution of *PON1* genotypes was not associated with anti-HCV positivity, used as a proxy for HCV infection susceptibility, in the HD population (Additional file 1: Table S4). However, when inheritance models were applied and adjusted for diabetic nephropathy and serum albumin concentration, the rs854560 AA + AT genotypes (*P* = 0.036) and the rs705379 CT + CC genotypes (*P* = 0.045) were more frequent in HCV susceptible individuals than the T genotype (Additional file 1: Table S5).

In individuals, who spontaneously resolved HCV infection, successful genotyping was obtained in 79 individuals for rs705379, 82—for rs854560, and 70—for rs662. In HCV RNA-positive individuals, the respective numbers were 99, 98, and 99.

LDs for *PON1* SNVs were as follows: rs662 and rs854560—D` 0.765, r^2^ 0.118, rs662 and rs705379—D` 0.001, r^2^ 0.000, and rs854560 and rs705379—D` 0.553, r^2^ 0.172. The LD analysis revealed r^2^ < 0.3 what indicates weak LD between tested *PON1* SNVs.

The rs662 genotype GG and allele G as well as the rs854560 genotype TT frequencies were higher in spontaneous HCV clearance individuals (Table 2). Concerning models of inheritance, *PON1* rs662 GG was associated with spontaneous HCV clearance in additive (*P*-value = 0.022) and recessive (*P*-value = 0.022) models, rs854560—in the recessive model (*P*-value = 0.003) (Additional file 1: Table S6).

**Table 2:** *PON1* polymorphic variants and spontaneous HCV clearance in HD individuals

Genotypes and alleles	Anti-HCV positive and HCV RNA negative	Anti-HCV and HCV RNA positive	Odds ratio (95% CI), *P*-value^a^
*PON1* rs662 n = 175, P-value for HWE = 0.295			
AA	37 (48.7%)	60 (60.6%)	Reference
AG	32 (42.1%)	38 (38.4%)	0.344
GG	7 (9.2%)	1 (1.0%)	0.009
Allele A	106 (69.7%)	158 (79.8%)	Reference
Allele G	46 (30.3%)	40 (20.2%)	0.034
*PON1* rs854560 n = 180, *P*-value for HWE = 0.440			
AA	31 (37.8%)	35 (35.7%)	Reference
AT	33 (40.2%)	57 (58.2%)	0.249
TT	18 (22%)	6 (6.1%)	0.030
Allele A	95 (57.9%)	127 (64.8%)	Reference
Allele T	69 (42.1%)	69 (35.2%)	0.193
*PON1* rs705379, n = 178, P-value for HWE = 0.582			
CC	21 (26.6%)	20 (20.2%)	Reference
CT	33 (41.8%)	52 (52.5%)	0.249
TT	25 (31.6%)	27 (27.3%)	0.836
Allele C	75 (47.5%)	92 (46.5%)	Reference
Allele T	83 (52.5%)	106 (53.5%)	0.915

^a^Fisher's exact test

The most common haplotype, rs662A_rs854560A, was inversely associated with spontaneous HCV clearance. Compared to this haplotype, the rs662G_rs854560T indicated a 5.1-fold higher odds for HCV clearance. However, when all three *PON1* variants were included in the haplotype analysis, there were no significant differences in the statistical analyses (Table 3).

**Table 3:** An analysis of haplotype *PON1* SNVs

SNVs	Haplotype	Freq	Case, control frequencies	Chi-square	*P*-value	*P*_corr_ value^a^	OR (95%CI)^b^, *P*-value	OR (95%CI)^c^, *P*-value
rs662_rs854560	AA	0.391	0.318, 0.452	6.628	0.010	0.019	0.57 (0.37–0.88), 0.011	Reference
	AT	0.363	0.391, 0.340	0.998	0.318	0.629	1.22 (0.79–1.88), 0.366	1.60 (0.99–2.59), 0.057
	GA	0.223	0.256, 0.196	1.812	0.178	0.374	1.40 (0.86–2.30), 0.179	1.83 (1.05–3.18), 0.031
	GT	0.022	0.035, 0.012	2.121	0.145	0.304	3.72 (0.74–18.7), 0.088	5.09 (0.99–26.17), 0.032
rs854560_rs705379	AC	0.392	0.372, 0.408	0.482	0.487	0.843	0.85 (0.56–1.31), 0.466	Reference
	TT	0.304	0.325, 0.286	0.611	0.435	0.803	1.22 (0.77–1.91), 0.396	1.26 (0.76–2.09), 0.365
	AT	0.223	0.202, 0.240	0.735	0.391	0.756	0.81 (0.49–1.34), 0.415	0.94 (0.54–1.63), 0.816
	TC	0.082	0.101, 0.066	1.508	0.219	0.491	1.54 (0.72–3.31), 0.263	1.64 (0.73–3.67), 0.225
rs662_rs854560_rs705379	ATT	0.286	0.297, 0.276	0.189	0.663	0.997	1.13 (0.71–1.79), 0.611	Reference
	AAC	0.277	0.235, 0.312	2.628	0.105	0.372	0.69 (0.43–1.11), 0.123	0.70 (0.40–1.23), 0.214
	GAC	0.115	0.138, 0.096	1.564	0.211	0.670	1.49 (0.77–2.85), 0.233	1.30 (0.63–2.69), 0.475
	AAT	0.114	0.082, 0.140	2.961	0.085	0.314	0.55 (0.28–1.11), 0.093	0.54 (0.25–1.17), 0.115
	GAT	0.109	0.119, 0.100	0.322	0.570	0.984	1.19 (0.61–2.31), 0.616	1.07 (0.51–2.24), 0.860
	ATC	0.078	0.095, 0.064	1.172	0.279	0.794	0.49 (0.17–1.39), 0.171	1.30 (0.56–3.00), 0.541
	GTT	0.018	0.028, 0.010	1.462	0.227	0.696	3.13 (0.60–16.4), 0.155	2.81 (0.52–15.2), 0.212

^a^*P*-value calculated using permutation test and a total of 1,000 permutations

^b^ ll other haplotypes pooled together were used as the reference

^c^The most common haplotype was used as the reference

The *IFNL4* rs368234815 TT/TT variant was equally distributed among individuals bearing different *PON1* SNVs (Additional file 1: Tables S2–S4). The epistatic gene–gene analysis did not reveal the interaction between tested *PON1* SNVs and *IFNL4* rs368234815 (*P* = 0.094, Table 4).

**Table 4:** Results of gene–gene interaction analysis

Genes and rs numbers	Testing Balanced Accuracy	Cross Validation Consistency	*P*-value^a^
*PON1*_rs854560, *IFNL4*_rs368234815	0.5874	7/10	0.206
*PON1*_rs662, *PON1*_rs854560, *IFNL4*_rs368234815	0.6069	10/10	0.094
*PON1*_rs662, *PON1*_rs854560, *PON1*_rs705379, *IFNL4*_rs368234815	0.5428	10/10	0.567

^a^Significance of accuracy, empirical p-value based on 1,000 permutations

The *PON1* rs662 GG genotype, *PON1* rs854560 TT genotype, *IFNL4* rs368234815 TT/TT genotype, age at RRT onset, RRT duration, and chronic glomerulonephritis were significantly associated with spontaneous HCV clearance in univariable analysis (Table 5). In multivariable analysis (Table 5), age at the RRT onset and chronic glomerulonephritis were no longer associated with spontaneous HCV clearance. The AUROC from the regression model was estimated at 0.768, 95% CI for AUC was 0.695–0.840 (Additional file 1: Fig. S1).

**Table 5:** The univariate and multivariate regression including possible predictors of spontaneous HCV clearance

Parameter	Level of effect	Odds ratio	Odds ratio Upper 95.0% CL	Odds ratio Lower 95.0% CL	*P*-value
The univariate regression analysis					
Age at the RRT onset (years)		1.019	1.004	1.034	0.014
RRT duration (years)		0.95	0.919	0.982	0.003
*PON1* rs854560 TT vs. AA + AT	TT	4.313	1.623	11.461	0.003
*PON1* rs662 GG vs. AA + AG	GG	9.800	1.179	81.451	0.035
*IFNL4* rs368234815 TT/TT vs. ∆G/TT + ∆G/∆G	TT/TT	2.600	1.427	4.734	0.002
Chronic glomerulonephritis	Yes	0.432	0.220	0.851	0.015
The multivariate regression analysis					
Age at the RRT onset (years)		0.990	0.965	1.016	0.452
RRT duration (years)		0.930	0.879	0.984	0.011
*PON1* rs854560 TT vs. AA + AT	TT	6.208	1.962	19.644	0.002
*PON1* rs662 GG vs. AA + AG	GG	10.762	1.222	94.796	0.032
*IFNL4* rs368234815 TT/TT vs. ∆G/TT + ∆G/∆G	TT/TT	2.607	1.298	5.235	0.007
Chronic glomerulonephritis	Yes	0.609	0.259	1.432	0.255

Hosmer Lemeshow goodness of fit *P* = 0. 949, Wald test *P* = 0.0001 for multivariate regression analysis

## Discussion

Our study showed that homozygosity of the *PON1* rs662 and rs854560 variant alleles (GG and TT, respectively) was associated with a higher frequency of spontaneous HCV clearance in HD patients. In the studied individuals, both these homozygotes appeared favorable with respect to HCV clearance in univariate and multivariate analyses.

Only variant homozygotes of *PON1* SNVs, located in the *PON1* coding region and associated with lower PON-1 activity/concentration (rs662 and rs854560), were associated with spontaneous HCV clearance. Inversely, by Ferré et al. [9], chronic hepatitis C was related to the *PON1* rs662 genotype involved in the generation of the PON-1 isoform, most able to hydrolyze paraoxon in this study. The haplotypes containing the favorable rs662 G allele and one of the rs854560 alleles were also associated with HCV clearance [9].

We initially assumed that associations of *PON1* SNVs with spontaneous HCV clearance could be related to their epistatic interaction with *IFNL4* rs368234815, which is the most powerful influencer of HCV outcome among genes of *IFNL3/4* region in HD individuals [34]. However, the analysis of epistatic gene–gene interaction between tested *PON1* SNVs and *IFNL4* rs368234815 did not show significance at a P-value below 0.05. *PON1* rs662, *PON1* rs854560, and *IFNL4* rs368234815 interacted at *P* = 0.094. Therefore, our findings were inconclusive as to whether or not an association was present. Additionally, it has to be mentioned that the *IFNL4* rs368234815 TT/TT variant was equally distributed among individuals bearing different *PON1* SNVs. Thus, none of the *PON1* SNVs had a higher frequency of the *IFNL4* rs368234815 TT/TT variant, which is strongly associated with spontaneous HCV clearance.

In non-uremic individuals, the circulating PON-1 activity and hydroperoxide levels are negatively correlated [40, 41]. HD individuals, who generally show lower serum PON-1 and arylesterase activities than healthy individuals [42, 43], did not present associations between *PON1* SNVs, PON-1 and arylesterase activities, plasma lipid peroxides, and biochemical parameters related to glutathione redox system [42, 44]. *PON1* rs662 and rs854560 haplotypes were also not associated with serum PON-1 activity [42]. In our earlier study, we determined serum PON-1 activity in 93 maintenance HD individuals [36]. Re-analysis of this group concerning HCV infection revealed six HCV RNA-positive individuals. They did not differ in serum PON-1 level from HCV RNA negative individuals (91.0 ± 23.0 U/L vs. 97.3 ± 38.5 U/l, *P* = 0.693). The latter group included one individual, which spontaneously eliminated HCV. Serum PON-1 activity does not seem to be a factor that differentiates HCV RNA-positive uremic HD individuals from the HCV RNA-negative.

It is puzzling why HD individuals, having *PON1* genotypes associated with lower serum PON-1 activity, more frequently clear HCV. Increased oxidative stress, hepatic inflammation, and lipid abnormalities related to acute HCV infection [45] provoke natural host defense mechanisms more intensively leading to HCV clearance. The higher PON-1 activities might be undesirable in conditions associated with acute HCV infection as they attenuate host reaction for HCV infection. Individuals who showed clinical symptoms of acute HCV infection were less likely to have the chronic liver disease [38, 46]. Severe acute infection causes a more vigorous immune response, resulting in higher HCV clearance and a lower rate of developing chronic infection [47].

To generalize our results, we suggest examining which phenotypes associated with *PON1* SNVs are responsible for HCV clearance and HCV infection susceptibility in further studies. Examinations are warranted to explore the possible interactions between SNVs associated with these newly discovered phenotypes and their genetic variants.

## Limitations

A primary limitation of our study is a relatively small number of HD individuals exposed to HCV infection. It can be a reason for which certain associations could have been underpowered. A limitation that affects the generalizability of our study is the Greater Poland voivodship region as the only one where investigations were performed.

Unfortunately, we do not have viral load and HCV genotypes in our database. HCV genotypes are not determined in dialysis centers, but hepatic units, and usually not transfer to nephrologists. Our individuals were not treated with antiviral medicines. Thus, they probably did not have HCV genotypes estimated. Further studies might have shown whether *PON1* SNV effects are associated with HCV viral load and genotypes.

## Conclusion

Our study on hemodialysis individuals shows associations between *PON1* SNVs and spontaneous HCV clearance (*PON1* rs662 GG and rs854560 TT genotypes are associated with higher odds of HCV clearance; the haplotype rs662G_rs854560T indicates higher odds of HCV clearance). The present data also indicate topics worth examining in more extensive studies as non-conclusive in our individuals (the epistatic gene–gene interaction between *PON1* SNVs and *IFNL4* rs368234815—*P* = 0.094) but in line with shown significant data concerning associations of *PON1* rs662 and *PON1* rs854560 with spontaneous HCV clearance. Similar results, performed in the general population, may show whether current findings are relevant only in HD individuals or are typical in non-uremic individuals.

## Supplementary Information

Below is the link to the electronic supplementary material.

**Additional file 1: Table S1.** A power analysis. **Table S2.** *PON1 *rs662 polymorphic variants and demographic, clinical, and laboratory data of HD patients (n = 1332). **Table S3.** *PON1 *rs854560 polymorphic variants and demographic, clinical, and laboratory data of HD patients (n = 1362). **Table S4.** *PON1 *rs705379 polymorphic variants and demographic, clinical, and laboratory data of HD patients (n = 1329). **Table S5.** Anti-HCV positivity concerning *PON1 *variants in HD patients. **Table S6.** *PON1 *polymorphic variants and spontaneous HCV clearance in HD individuals. **Fig. S1.** The ROC curve for the multivariate regression model.

## Data Availability

The datasets used and/or analyzed during the current study are available from the corresponding author on reasonable request.
