# Supplementary material for: Paraoxonase 1 gene (PON1) variants concerning hepatitis C virus (HCV) spontaneous clearance in hemodialysis individuals: a case–control study
Source: BMC Infect Dis. 2021 Aug 26;21:875. doi: 10.1186/s12879-021-06597-4 (PMC8394142; doi:10.1186/s12879-021-06597-4)
Supplement: Supplementary file 1 — Additional file 1: Table S1. A power analysis. Table S2. PON1 rs662 polymorphic variants and demographic, clinical, and laboratory data of HD patients (n = 1332). Table S3. PON1 rs854560 polymorphic variants and demographic, clinical, and laboratory data of HD patients (n = 1362). Table S4. PON1 rs705379 polymorphic variants and demographic, clinical, and laboratory data of HD patients (n = 1329). Table S5. Anti-HCV positivity concerning PON1 variants in HD patients. Table S6. PON1 polymorphic variants and spontaneous HCV clearance in HD individuals. Fig. S1. The ROC curve for the multivariate regression model. [file 12879_2021_6597_MOESM1_ESM.docx]

Additional file 1

Additional file 1 Table S1. A power analysis

| - Cases = 100 |  |
| --- | --- |
| - Controls = 100 | |
| Prevalence = 42% | |
| Significance level = 0.05 | |

|  |  |  | **EXPECTED POWER** | | |
| --- | --- | --- | --- | --- | --- |
| **Nucleotide** | **Disease allele** | **Genotype** | **Disease model** | | |
| **variant** | **frequency^a^** | **Relative Risk** | **Additive** | **Dominant** | **Recessive** |
| **rs662** | 0.2815 | 1.00 | 0.050 | 0.050 | 0.050 |
|  |  | 1.25 | 0.395 | 0.236 | 0.083 |
|  |  | 1.50 | 0.857 | 0.610 | 0.182 |
|  |  | 1.75 | 0.984 | 0.866 | 0.338 |
|  |  | 2.00 | 0.999 | 0.965 | 0.523 |
|  |  | 2.25 | 1.000 | 0.992 | 0.698 |
|  |  | 2.50 | 1.000 | 0.998 | 0.833 |
| **rs854560** | 0.3660 | 1.00 | 0.050 | 0.050 | 0.050 |
|  |  | 1.25 | 0.419 | 0.208 | 0.114 |
|  |  | 1.50 | 0.863 | 0.527 | 0.296 |
|  |  | 1.75 | 0.983 | 0.781 | 0.545 |
|  |  | 2.00 | 0.998 | 0.913 | 0.767 |
|  |  | 2.25 | 1.000 | 0.967 | 0.906 |
|  |  | 2.50 | 1.000 | 0.988 | 0.970 |
| **rs705379** | 0.4803 | 1.00 | 0.050 | 0.050 | 0.050 |
|  |  | 1.25 | 0.414 | 0.157 | 0.165 |
|  |  | 1.50 | 0.836 | 0.376 | 0.461 |
|  |  | 1.75 | 0.969 | 0.588 | 0.760 |
|  |  | 2.00 | 0.994 | 0.742 | 0.926 |
|  |  | 2.25 | 0.999 | 0.840 | 0.984 |
|  |  | 2.50 | 1.000 | 0.901 | 0.997 |

^a^ population European (Non-Finnish) - the Genome Aggregation Database (gnomAD v3.1.1)

Additional file 1 Table S2. *PON1* rs662 polymorphic variants and demographic, clinical, and laboratory data of HD patients (n = 1332)

| Parameter | *PON1* rs662  Genotype AA  n = 729 | *PON1* rs662  Genotype AG  n = 493 | *PON1* rs662  Genotype GG  n = 110 | P value^†^ |
| --- | --- | --- | --- | --- |
| Male gender | 413 (56.6%) | 278 (56.2%) | 54 (49.1%) | 0.332 |
| Age at RRT onset, years | 59.6 (7.2 – 91.7) | 61 (7.7 – 87.6) | 61.8 (11.8 – 86.6) | 0.856 |
| Diabetic nephropathy | 213 (29.2%) | 138 (28%) | 25 (22.7%) | 0.377 |
| Chronic glomerulonephritis | 119 (16.3%) | 70 (14.2%) | 17 (15.5%) | 0.610 |
| Hypertensive nephropathy | 132 (18.1%) | 108 (21.9%) | 22 (20%) | 0.247 |
| RRT duration, years | 5.9 (0.02 – 34.01) | 5.9 (0.07 – 30.29) | 5.58 (0.32 – 24.47) | 0.853 |
| BMI, kg/m^2^ | 25.16 (14.33 – 51.02) | 25.45 (15.4 – 59.17) | 25.76 (16.42 – 41.62) | 0.716 |
| HBsAg positivity | 37 (5.1%) | 18 (3.7%) | 1 (0.9%) | 0.092 |
| Anti-HCV positive | 97 (13.3%) | 70 (14.1%) | 8 (7.3 %) | 0.142 |
| ALT, IU/L | 14 (0.6 – 195) | 13 (2 – 135) | 15 (4 – 131) | 0.503 |
| AST, IU/L | 16 (3 – 177) | 15 (5 – 115.4) | 15 (4 – 97) | 0.316 |
| ALP, IU/L | 98.29 (12.25 – 1408) | 97.13 (19 – 1684) | 95.75 (13.5 – 713.5) | 0.389 |
| GGT, IU/L | 29 (4 – 692) | 28 (1 – 682) | 29 (5 – 148) | 0.769 |
| C-reactive protein, mg/L | 5.7 (0 – 198.6) | 6 (0.2 – 247.1) | 6 (0.4 – 135.7) | 0.683 |
| Albumin, g/dL | 4 (1.9 – 41) | 3.9 (1.9 – 68) | 4 (2.8 – 42) | 0.800 |
| Platelet count, G/L | 193 (44 – 457) | 183 (41 – 450) | 224 (60 – 383) | 0.219 |
| Total cholesterol (mg/dL) | 168.5 (71.5 – 626) | 172 (51 – 368) | 170.1 (84 – 336) | 0.904 |
| HDL cholesterol (mg/dL) | 40 (5 – 146.8) | 39 (8 – 103) | 42 (12 – 103) | 0.105 |
| LDL cholesterol (mg/dL) | 94 (17.4 – 512) | 97 (13.3 – 369) | 96.8 (33 – 215) | 0.506 |
| TG (mg/dL) | 148 (32 – 856) | 144 (35 – 1363) | 147.4 (29.8 – 1105) | 0.732 |
| Non-HDL-cholesterol (mg/dL) | 125 (8 – 593) | 131 (32 – 329) | 127 (42.1 – 296) | 0.643 |
| LDL/HDL cholesterol ratio | 0.65 (0.04 – 2.88) | 0.67 (0.06 – 4.6) | 0.66 (0.06 – 1.82) | 0.681 |
| HDL/TC ratio | 0.24 (0.05 – 0.91) | 0.23 (0.09 – 0.59) | 0.25 (0.1 – 0.56) | 0.165 |
| TG/HDL cholesterol ratio | 3.61 (0.44 – 49.71) | 3.62 (0.47 – 32.45) | 3.5 (0.66 – 34.53) | 0.455 |
| TG/HDL-cholesterol ratio ≥3.8 | 315 (48%) | 207 (47.2%) | 44 (43.6%) | 0.707 |
| *IFNL4* rs368234815 TT/TT, n = 195 | 116 (45.1% of 257) | 67 (42.4% of 158) | 12 (48% of 25) | 0.797 |

Results are presented as median and range (minimum-maximum) or the number of patients presenting the indicated parameter with the % of the total of tested patients shown in parentheses.

† – Kruskal-Wallis test for quantitative variables, Fisher’s 3x2 exact test for quantitative variables

Conversion factors to SI units are as follows: for alanine aminotransferase – 1 IU/L = 0.0167 µkat/L, for albumin – 1 g/dL = 10 g/L, for alkaline phosphatase – 1 IU/L = 0.0167 µkat/L, for aspartate aminotransferase – 1 IU/L = 0.0167 µkat/L, for C-reactive protein – 1 mg/L = 9.524 nmol/L, for gamma-glutamyltransferase – 1 IU/L = 0.0167 µkat/L

Abbreviations: ALP - alkaline phosphatase, ALT - alanine aminotransferase, AST - aspartate aminotransferase, Anti-HCV – antibodies against hepatitis C virus, BMI *–* body mass index, GGT - gamma-glutamyltransferase, HBsAg – surface antigen of hepatitis B virus, HCV –hepatitis C virus, HD – hemodialysis, RRT – renal replacement therapy

Additional file 1 Table S3. *PON1* rs854560 polymorphic variants and demographic, clinical, and laboratory data of HD patients (n = 1362)

| Parameter | *PON1* rs854560  Genotype AA  n = 581 | *PON1* rs854560  Genotype AT  n = 622 | *PON1* rs854560  Genotype TT  n = 159 | P value^†^ |
| --- | --- | --- | --- | --- |
| Male gender | 307 (52.7%) | 355 (57%) | 93 (58.5%) | 0.221 |
| Age at RRT onset, years | 60.4 (11.8 – 89.4) | 61.4 (7.7 – 91.7) | 59.2 (7.2 – 90.1) | 0.477 |
| Diabetic nephropathy | 151 (26%) | 189 (30.3%) | 50 (31.4%) | 0.168 |
| Chronic glomerulonephritis | 96 (16.5%) | 92 (14.8%) | 22 (13.8%) | 0.619 |
| Hypertensive nephropathy | 112 (19.3%) | 128 (20.5%) | 28 (17.6%) | 0.700 |
| RRT duration, years | 5.78 (0.07 – 32.81) | 6.09 (0.04 – 34.01) | 5 (0.02 – 30.07) | 0.151 |
| BMI, kg/m^2^ | 25.24 (15.4 – 51.02) | 25.66 (14.33 – 59.17) | 24.89 (17.86 – 35.98) | 0.491 |
| HBsAg positivity | 18 (3.1%) | 28 (4.6%) | 10 (6.3%) | 0.153 |
| Anti-HCV positive | 66 (11.3%) | 90 (14.4%) | 24 (15.1%) | 0.196 |
| ALT, IU/L | 14 (2 – 195) | 14 (1 – 107) | 14 (0.6 – 45) | 0.582 |
| AST, IU/L | 15 (4 – 152) | 15 (3 – 177) | 16 (3 – 63) | 0.680 |
| ALP, IU/L | 97 (25.75 – 1299.33) | 99.25 (19 – 1684) | 94.5 (12.25 – 1109.5) | 0.304 |
| GGT, IU/L | 29 (5 – 692) | 29 (1 – 565) | 27 (4 – 443) | 0.604 |
| C-reactive protein, mg/L | 6 (0.1 – 198.6) | 5.7 (0 – 247.1) | 5.3 (0.3 – 78.6) | 0.852 |
| Albumin, g/dL | 4 (1.9 – 42) | 3.9 (1.9 – 68) | 4 (2 – 41) | 0.570 |
| Platelet count, G/L | 207 (41 – 501) | 183 (56 – 450) | 196.5 (76 – 457) | 0.239 |
| Total cholesterol (mg/dL) | 169 (65 – 626) | 170 (51 – 368) | 172 (85 – 337) | 0.808 |
| HDL cholesterol (mg/dL) | 40.2 (6 – 118) | 40 (7 – 146.8) | 40 (5 – 92) | 0.193 |
| LDL cholesterol (mg/dL) | 94.4 (17 – 512) | 95.3 (13.3 – 369) | 99.7 (35 – 239.3) | 0.645 |
| TG (mg/dL) | 144 (29.8 – 1105) | 144 (32 – 1363) | 155 (36.3 – 856) | 0.461 |
| Non-HDL-cholesterol (mg/dL) | 127 (40 – 593) | 128 (8 – 329) | 129.5 (58 – 299) | 0.603 |
| LDL/HDL cholesterol ratio | 0.67 (0.06 – 4.6) | 0.65 (0.04 – 3.3) | 0.6 (0.14 – 2.37) | 0.750 |
| HDL/TC ratio | 0.24 (0.05 – 0.62) | 0.24 (0.06 – 0.91) | 0.22 (0.06 – 0.61) | 0.057 |
| TG/HDL cholesterol ratio | 3.48 (0.44 – 34.53) | 3.63 (0.47 – 49.71) | 3.91 (0.65 – 39.4) | 0.124 |
| TG/HDL-cholesterol ratio ≥3.8 | 228 (43.9%) | 274 (48.6%) | 74 (51.7%) | 0.148 |
| *IFNL4* rs368234815 TT/TT, n = 197 | 73 (42.9%) | 96 (42.9%) | 28 (50.9%) | 0.537 |

Results are presented as median and range (minimum-maximum) or the number of patients presenting the indicated parameter with the % of the total of tested patients shown in parentheses.

† – Kruskal-Wallis test for quantitative variables, Fisher’s 3x2 exact test for quantitative variables

Conversion factors to SI units are as follows: for alanine aminotransferase – 1 IU/L = 0.0167 µkat/L, for albumin – 1 g/dL = 10 g/L, for alkaline phosphatase – 1 IU/L = 0.0167 µkat/L, for aspartate aminotransferase – 1 IU/L = 0.0167 µkat/L, for C-reactive protein – 1 mg/L = 9.524 nmol/L, for gamma-glutamyltransferase – 1 IU/L = 0.0167 µkat/L

Abbreviations: ALP - alkaline phosphatase, ALT - alanine aminotransferase, AST - aspartate aminotransferase, Anti-HCV – antibodies against hepatitis C virus, BMI *–* body mass index, GGT - gamma-glutamyltransferase, HBsAg – surface antigen of hepatitis B virus, HCV –hepatitis C virus, HD – hemodialysis, RRT – renal replacement therapy

Additional file 1 Table S4. *PON1* rs705379 polymorphic variants and demographic, clinical, and laboratory data of HD patients (n = 1329)

| Parameter | *PON1* rs705379  Genotype CC  n = 380 | *PON1* rs705379  Genotype CT  n = 641 | *PON1* rs705379  Genotype TT  n = 308 | P value^†^ |
| --- | --- | --- | --- | --- |
| Male gender | 215 (56.4%) | 351 (54.6%) | 171 (55.5%) | 0.855 |
| Age at RRT onset, years | 60.6 (7.2 – 91) | 60.2 (7.7 – 91.7) | 61.7 (8.7 – 90.1) | 0.164 |
| Diabetic nephropathy | 104 (27.4%) | 168 (26.2%) | 106 (34.4%) | 0.029^#^ |
| Chronic glomerulonephritis | 66 (17.4%) | 94 (14.6%) | 45 (14.6%) | 0.464 |
| Hypertensive nephropathy | 66 (17.4%) | 127 (19.8%) | 65 (21.1%) | 0.442 |
| RRT duration, years | 6.23 (0.19 – 32.81) | 6.08 (0.04 – 34.01) | 5.16 (0.02 – 32.61) | 0.047^$^ |
| BMI, kg/m^2^ | 25.19 (15.7 – 51.02) | 25.44 (14.33 – 59.17) | 25.57 (15.58 – 44.8) | 0.464 |
| HBsAg positivity | 17 (4.5%) | 29 (4.6%) | 9 (3.0%) | 0.495 |
| Anti-HCV positive | 41 (10.8%) | 85 (13.2%) | 52 (16.9%) | 0.066 |
| ALT, IU/L | 15 (2 – 195) | 14 (1 – 135) | 13 (0.6 – 120.3) | 0.236 |
| AST, IU/L | 15 (4 – 152) | 15 (3 – 139) | 16 (3 – 177) | 0.428 |
| ALP, IU/L | 98.5 (24 – 1299.33) | 97.25 (12.25 – 1684) | 97.75 (34.5 – 1353.25) | 0.773 |
| GGT, IU/L | 29 (5 – 593) | 28 (1 – 692) | 29 (6 – 682) | 0.965 |
| C-reactive protein, mg/L | 6 (0 – 195) | 5.8 (0.1 – 247.1) | 5.3 (0.1 – 142) | 0.536 |
| Albumin, g/dL | 4 (2 – 41) | 3.9 (1.9 – 68) | 3.9 (2 – 40) | 0.029^&^ |
| Platelet count, G/L | 191.5 (44 – 380) | 189 (41 – 501) | 198 (70 – 457) | 0.864 |
| Total cholesterol (mg/dL) | 170.5 (75 – 336) | 170 (65 – 626) | 167.7 (51 – 363) | 0.608 |
| HDL cholesterol (mg/dL) | 41 (12 – 103) | 40 (6 – 146.8) | 39 (5 – 92) | 0.138 |
| LDL cholesterol (mg/dL) | 97 (20 – 223) | 94.6 (17.4 – 512) | 94 (13.3 – 369) | 0.536 |
| TG (mg/dL) | 142 (39 – 1105) | 143 (29.8 – 1363) | 157 (32 – 856) | 0.275 |
| Non-HDL-cholesterol (mg/dL) | 128 (27 – 296) | 128 (8 – 593) | 125.3 (32 – 313) | 0.973 |
| LDL/HDL cholesterol ratio | 0.68 (0.06 – 2.07) | 0.66 (0.06 – 4.6) | 0.65 (0.04 – 3.3) | 0.119 |
| HDL/TC ratio | 0.24 (0.11 – 0.64) | 0.24 (0.05 – 0.91) | 0.23 (0.06 – 0.61) | 0.562 |
| TG/HDL cholesterol ratio | 3.42 (0.66 – 34.53) | 3.57 (0.44 – 49.71) | 3.92 (0.65 – 39.4) | 0.085 |
| TG/HDL-cholesterol ratio ≥3.8 | 147 (43.1%) | 266 (46.2%) | 145 (51.8%) | 0.093 |
| *IFNL4* rs368234815 TT/TT, n = 192 | 54 (44.6%) | 87 (42.4%) | 51 (44.7%) | 0.901 |

Results are presented as median and range (minimum-maximum) or the number of patients presenting the indicated parameter with the % of the total of tested patients shown in parentheses.

† – Kruskal-Wallis test for quantitative variables, Fisher’s 3x2 exact test for quantitative variables

Post hoc tests: # - significant between CT and TT genotypes (P = 0.029); $ - not significant between genotypes, & - significant between CC and TT genotypes (P = 0.029)

Conversion factors to SI units are as follows: for alanine aminotransferase – 1 IU/L = 0.0167 µkat/L, for albumin – 1 g/dL = 10 g/L, for alkaline phosphatase – 1 IU/L = 0.0167 µkat/L, for aspartate aminotransferase – 1 IU/L = 0.0167 µkat/L, for C-reactive protein – 1 mg/L = 9.524 nmol/L, for gamma-glutamyltransferase – 1 IU/L = 0.0167 µkat/L

Abbreviations: ALP - alkaline phosphatase, ALT - alanine aminotransferase, AST - aspartate aminotransferase, Anti-HCV – antibodies against hepatitis C virus, BMI *–* body mass index, GGT - gamma-glutamyltransferase, HBsAg – surface antigen of hepatitis B virus, HCV – hepatitis C virus, HD – hemodialysis, *PON1* – paraoxonase 1 gene, RRT – renal replacement therapy

Additional file 1 Table S5. Anti-HCV positivity concerning *PON1* variants in HD patients

| Genotypes | Anti-HCV positive patients | Anti-HCV negative patients  (Reference) | Odds ratio (95% CI),  P-value^a^ |
| --- | --- | --- | --- |
| *PON1* rs662 (575A>G) n = 1335, P-value for HWE = 0.046 | | | |
| AA vs. AG vs. GG | 98 (55.7%) vs. 70 (39.8%) vs. 8 (4.5%) | 632 (54.5%) vs. 425 (36.7%) vs. 102 (8.8%) | 0.299^b^ |
| AG + GG vs. AA | 78 (44.3%) vs. 98 (55.7%) | 527 (45.5%) vs. 632 (54.5%) | 0.954 (0.694, 1.313)  0.808 (0.305)^c^ |
| GG vs. AA + AG | 8 (4.5%) vs. 168 (95.5%) | 102 (8.8%) vs. 1057 (91.2%) | 0.493 (0.236, 1.032) 0.056 (0.105)^c^ |
| *PON1* rs854560 (163A>T) n = 180, P-value for HWE = 0.440 | | | |
| TT vs. AT vs. AA | 66 (36.7%) vs. 90 (50%) vs. 24 (13.3%) | 517 (43.6%) vs. 533 (45%) vs. 135 (11.4%) | 0.096^b^ |
| AT + TT vs. AA | 156 (86.7%) vs. 24 (13.3%) | 1050 (88.6%) vs. 135 (11.4%) | 0.836 (0.525, 1.331)  0.455 (0.490)^c^ |
| TT vs. AA + AT | 66 (36.7%) vs. 114 (63.3%) | 517 (43.6%) vs. 668 (56.4%) | 0.748 (0.541, 1.034)  0.089 (0.036)^c^ |
| *PON1* rs705379 (−108C>T), n = 1332, P-value for HWE = 0.248 | | | |
| TT vs. CT vs. CC | 41 (23%) vs. 85 (47.8%) vs. 52 (29.2%) | 340 (29.5%) vs. 558 (48.4%) vs. 256 (22.2%) | 0.020^b^ |
| CT + TT vs. CC | 137 (77%) vs. 41 (23%) | 814 (70.5%) vs. 340 (29.5%) | 1.396 (0.963, 2.023)  0.090 (0.429)^c^ |
| TT vs. CT + CC | 52 (29.2%) vs. 126 (70.8%) | 256 (22.2%) vs. 898 (77.8%) | 1.448 (1.018, 2.058)  0.045 (0.638)^c^ |

a - Fisher's exact test

b - Cochran-Armitage test

c - P-value for adjustment for diabetic nephropathy and serum albumin concentration

Additional file 1 Table S6. *PON1* polymorphic variants and spontaneous HCV clearance in HD individuals

| Genotypes | Anti-HCV positive and HCV RNA negative | Anti-HCV and HCV RNA positive  (Reference) | Odds ratio (95% CI),  P-value^a^ |
| --- | --- | --- | --- |
| *PON1* rs662 (575A>G) n = 175, P-value for HWE = 0.295 | | | |
| AA vs. AG vs. GG | 37 (48.7%) vs. 32 (42.1%) vs. 7 (9.2%) | 60 (60.6%) vs. 38 (38.4%) vs. 1 (1.0%) | 0.022^b^  GG vs. AA: 11.36, (1.342, 100.00), 0.009^c^ |
| AG + GG vs. AA | 39 (51.3%) vs. 37 (48.7%) | 39 (39.4%) vs. 60 (60.6%) | 1.622 (0.886, 2.967) 0.127 |
| GG vs. AA + AG | 7 (9.2%) vs. 69 (90.8%) | 1 (1.0%) vs. 98 (99.0%) | 9.942 (1.196, 82.65) 0.022 |
| *PON1* rs854560 (163A>T) n = 180, P-value for HWE = 0.440 | | | |
| AA vs. AT vs. TT | 31 (37.8%) vs. 33 (40.2%) vs. 18 (22.0%) | 35 (35.7%) vs. 57 (58.2%) vs. 6 (6.1%) | 0.169^b^ |
| AT + TT vs. AA | 51 (62.2%) vs. 31 (37.8%) | 63 (64.3%) vs. 35 (35.7%) | 0.914 (0.497, 1.679)  0.877 |
| TT vs. AA + AT | 18 (22.0%) vs. 64 (78.0%) | 6 (6.1%) vs. 92 (93.9%) | 4.313 (1.623, 11.462) 0.003^d^ |
| *PON1* rs705379 (−108C>T), n = 178, P-value for HWE = 0.582 | | | |
| TT vs. CT vs. CC | 21 (26.6%) vs. 33 (41.8%) vs. 25 (31.6%) | 20 (20.2%) vs. 52 (52.5%) vs. 27 (27.3%) | 0.853^b^ |
| CT + TT vs. CC | 58 (73.4%) vs. 21 (26.6%) | 79 (79.8%) vs. 20 (20.2%) | 0.699 (0.347, 1.408)  0.371 (0.638)^e^ |
| TT vs. CT + CC | 25 (31.6%) vs. 54 (68.4%) | 27 (27.3%) vs. 72 (72.7%) | 1.235 (0.646, 2.361)  0.691 (0.429)^e^ |

a - Fisher's exact test

b - Cochran-Armitage test

c – The analysis showing which genotypes differ in their frequencies between both study groups

d – Significant after the Bonferroni correction (P-value <0.006)

e – P-value for adjustment for diabetic nephropathy and serum albumin concentration

Additional file 1 Fig. S1. The ROC curve for the multivariate regression model

The model includes the *PON1* rs662 GG genotype, the *PON1* rs854560 TT genotype, the *IFNL4* rs368234815 TT/TT genotype, age at RRT onset, RRT duration, and chronic glomerulonephritis as possible explanatory variables for spontaneous HCV clearance.
